# Increasing stomatal CO_2_ conductance as a potential mechanism of photosynthetic activation by electrical signals in terrestrial plants

**DOI:** 10.3389/fpls.2024.1476175

**Published:** 2024-11-07

**Authors:** Vladimir Sukhov

**Affiliations:** Department of Biophysics, N. I. Lobachevsky State University of Nizhny Novgorod, Nizhny Novgorod, Russia

**Keywords:** electrical signals, photosynthetic activation, photosynthetic inactivation, stomatal CO_2_ conductance, mesophyll CO_2_ conductance, terrestrial plants

## 1 Introduction

Terrestrial plants are affected by action of numerous stressors (e.g., increased or decreased temperatures, excess light, mechanic damages, and others) which can be spatially heterogenous. Induction of physiological responses in non-irritated parts of plant can be based on generation and propagation of electrical signals (ESs) including action potentials, variation potentials, and system potentials (Gallé et al., 2015; Li et al., 2021; Pachú et al., 2021; Sukhova et al., 2023). It is known (Gallé et al., 2015; Farmer et al., 2020; Li et al., 2021) that ESs influence expression of defense genes, production of phytohormones, respiration, phloem mass-flow, transpiration, and many other processes and, thereby, increase plant tolerance to action of adverse factors (Szechyńska-Hebda et al., 2017; Zandalinas et al., 2020).

Photosynthesis is an important target of ESs because these signals can change the CO_2_ assimilation (A_hv_), quantum yields of photosystem I and II (γ_PSI_ and γ_PSII_), non-photochemical quenching of chlorophyll fluorescence (NPQ), linear electron flow (LEF), and cyclic electron flow around photosystem I (CEF) (Gallé et al., 2015; Szechyńska-Hebda et al., 2017; Sukhova et al., 2023). It is traditionally considered that ESs suppress photosynthetic processes decreasing A_hv_, γ_PSI_, γ_PSII_, and LEF and increasing NPQ and CEF (see, e.g., Lautner et al., 2005; Grams et al., 2009; Pavlovič et al., 2011; Gallé et al., 2013; Sukhov et al., 2015; Białasek et al., 2017; Krausko et al., 2017; Szechyńska-Hebda et al., 2022). However, there are works which show that electrical signals can activate photosynthesis including increasing A_hv_ and γ_PSII_ (Grams et al., 2007; Vuralhan-Eckert et al., 2018; Yudina et al., 2022; Grinberg et al., 2023). Considering an important role of the ESs-induced photosynthetic inactivation in stimulation of the plant tolerance to adverse factors (Sukhov, 2016; Sukhova et al., 2023), potential mechanisms of the positive influence of electrical signals on photosynthesis require additional discussion.

## 2 Mechanisms of ESs-induced photosynthetic inactivation

Mechanisms of the ESs-induced photosynthetic inactivation are relatively investigated now (Sukhov, 2016; Sukhova et al., 2023). There are at least two groups of potential targets of electrical signals: photosynthetic dark and light reactions (Pavlovič et al., 2011; Gallé et al., 2013; Sukhov et al., 2015). The ESs influence on both targets is related to transient inactivation of H^+^-ATPase in the plasma membrane, which accompanies induction of all types of ESs, increases pH in the apoplast, and decreases pH in the cytoplasm, stroma and lumen of chloroplasts (Sukhova et al., 2023).

Decreasing pH in the chloroplast lumen inactivates photosynthetic light reactions through the direct NPQ stimulation by the PsbS protonation (Ruban, 2015, 2016) and LEF suppression by slowing the plastoquinol oxidation (Tikhonov, 2013, 2014); the last process activates CEF (Sukhov et al., 2015). In contrast, the ESs-induced inactivation of photosynthetic dark reactions is caused by disruption of the CO_2_ flux into the chloroplast stroma through decreasing the mesophyll CO_2_ conductance (g_m_) (Gallé et al., 2013). This response is probable to be related to pH-dependent increase of HCO_3_^-^: CO_2_ ratio in the apoplast (Sukhova et al., 2023) because HCO_3_^-^ is weakly transported through the plasma membrane (Tholen and Zhu, 2011). Alternative hypotheses explain influence of pH shifts on g_m_ through changes in activity of carbonic anhydrases (Grams et al., 2009) or aquaporins (Gallé et al., 2013). It should be noted that the ESs-induced inactivation of photosynthetic dark reactions also suppresses photosynthetic light reactions providing additional mechanism of changes in NPQ, γ_PSI_, γ_PSII_, LEF, and CEF (Pavlovič et al., 2011; Sukhov et al., 2015; Sukhov, 2016).

## 3 Participation of changes in stomatal CO_2_ conductance in forming ESs-induced photosynthetic activation and inactivation

It is known (Flexas et al., 2008, 2012) that the CO_2_ flux from air to the chloroplast stroma is dependent on g_m_ and the stomatal CO_2_ conductance (g_s_). Potentially, it means that changes in g_s_ can also participate in induction of photosynthetic responses by electrical signals. However, ESs-induced decreasing the CO_2_ assimilation is accompanied by increasing the stomatal CO_2_ conductance in many investigations (Koziolek et al., 2004; Grams et al., 2009; Gallé et al., 2013); therefore, the changes in g_s_ can weakly influence A_hv_ in some cases.

Assuming that g_m_ and g_s_ are series connected (Flexas et al., 2008), (Equation 1) can be used for description of the total CO_2_ conductance (g):


(1) $$\text{g}=\frac{{\text{g}}_{\text{s}}{\text{g}}_{\text{m}}}{{\text{g}}_{\text{s}}+{\text{g}}_{\text{m}}}$$

Equation 1 shows that g≈g_m_ at g_s_>>g_m_; i.e., ESs-induced changes in g_s_ should not influence the total CO_2_ conductance and, therefore, assimilation in this case. This point is in a good accordance with works noted above. In contrast, ESs-induced changes in g_s_ can influence g and, thereby, A_hv_ at g_s_≈g_m_ or g_m_>>g_s_; in the last case (g_m_>>g_s_), decreasing g_m_ should not strongly influence the photosynthetic CO_2_ assimilation.

It is known that electrical signals can induce both initial increasing and decreasing g_s_ (Koziolek et al., 2004; Kaiser and Grams, 2006; Grams et al., 2007, 2009; Gallé et al., 2013). Increasing g_s_ can be observed under specific conditions; e.g., under the high air humidity (Yudina et al., 2019) or strong soil drought (Yudina et al., 2022). It is also probable that ESs induce this increasing in some plant species including, e.g., maize (Grams et al., 2009), mimosa (Kaiser and Grams, 2006), and soybean (Gallé et al., 2013). In accordance with Yudina et al. (2019), final ESs-induced changes in stomatal conductance can be caused by combination of two opposite processes: decreasing turgor of guard cells, which contributes the stomata closure, and decreasing turgor of epidermal cells, which contributes the stomata opening. Therefore, different contributions of these mechanisms can provide both opening and closure of stomata. The turgor decreasing is related to fluxes of ions and protons from the cytoplasm to apoplast, which accompany the transient inactivation of H^+^-ATPase and changes in activity of ion channels during the ESs generation (Sukhov, 2016; Sukhova et al., 2023).

As a result, it can be hypothesized that the positive influence of ESs on photosynthesis requires increasing g_s_ and substantial contribution of g_s_ to g (g_m_>>g_s_). Despite absence of works, which directly investigated influence of ESs-induced increasing g_s_ on A_hv_ under the low g_s_:g_m_ ratio, there are points supporting this hypothesis. First, the ESs-induced activation of the photosynthetic CO_2_ assimilation has dynamics being similar to dynamics of increasing g_s_ in all investigations (Peña-Cortés et al., 1995; Grams et al., 2007; Vuralhan-Eckert et al., 2018; Yudina et al., 2022; Grinberg et al., 2023); in contrast, dynamics of the A_hv_ inactivation can be similar (Hlavácková et al., 2006; Hlavinka et al., 2012) or different (Koziolek et al., 2004; Kaiser and Grams, 2006; Grams et al., 2009; Gallé et al., 2013) with dynamics of decreasing g_s_ in various works.

Second, decreasing the initial g_s_ (under the strong water deficit), which increases contribution of g_s_ to g in accordance with (Equation 1), transforms the negative influence of electrical signals on photosynthesis into the positive influence (Yudina et al., 2022). In this case, magnitude of the ESs-induced increasing A_hv_ is positively dependent on the magnitude of the ESs-induced g_s_ increasing. In contrast, decreasing the initial g_m_ (under the high CO_2_ concentration) increases magnitude of the ESs-induced inactivation of photosynthetic CO_2_ assimilation (Gallé et al., 2013). It should be additionally noted that pea seedlings, which mainly demonstrate unrelated dynamics of changes in g_s_ and A_hv_ under favorable conditions (Yudina et al., 2019, 2022), have the high g_s_:g_m_ ratio under these conditions (Sukhov et al., 2017); i.e., the total CO_2_ flux into the chloroplast stroma is limited by g_m_ in this plant.

It should be additionally noted that combination of the ESs-induced increasing g_s_ and similar initial values of g_m_ and g_s_ (g_s_≈g_m_) can potentially contribute to intricate dynamics of changes in photosynthetic activity because decreasing g_m_ should suppress the CO_2_ assimilation; in contrast, increasing g_s_ should stimulate this assimilation. Peña-Cortés et al. (1995) showed intricate dynamics of photosynthetic changes (including increasing and decreasing A_hv_) after local action of various stressors (the electrical current, mechanical damage, heating); however, these changes were rather related to the intricate dynamics of g_s_ after irritations.

Thus, simultaneous presence of both noted properties (the ESs-induced increasing g_s_ and substantial contribution of g_s_ to g) seems to be possible; at least, in some plants and under specific conditions. It can explain variety of direction of ESs-induced photosynthetic responses (activation or inactivation) which were shown in various works.

## 4 Discussion

**Figure 1:**
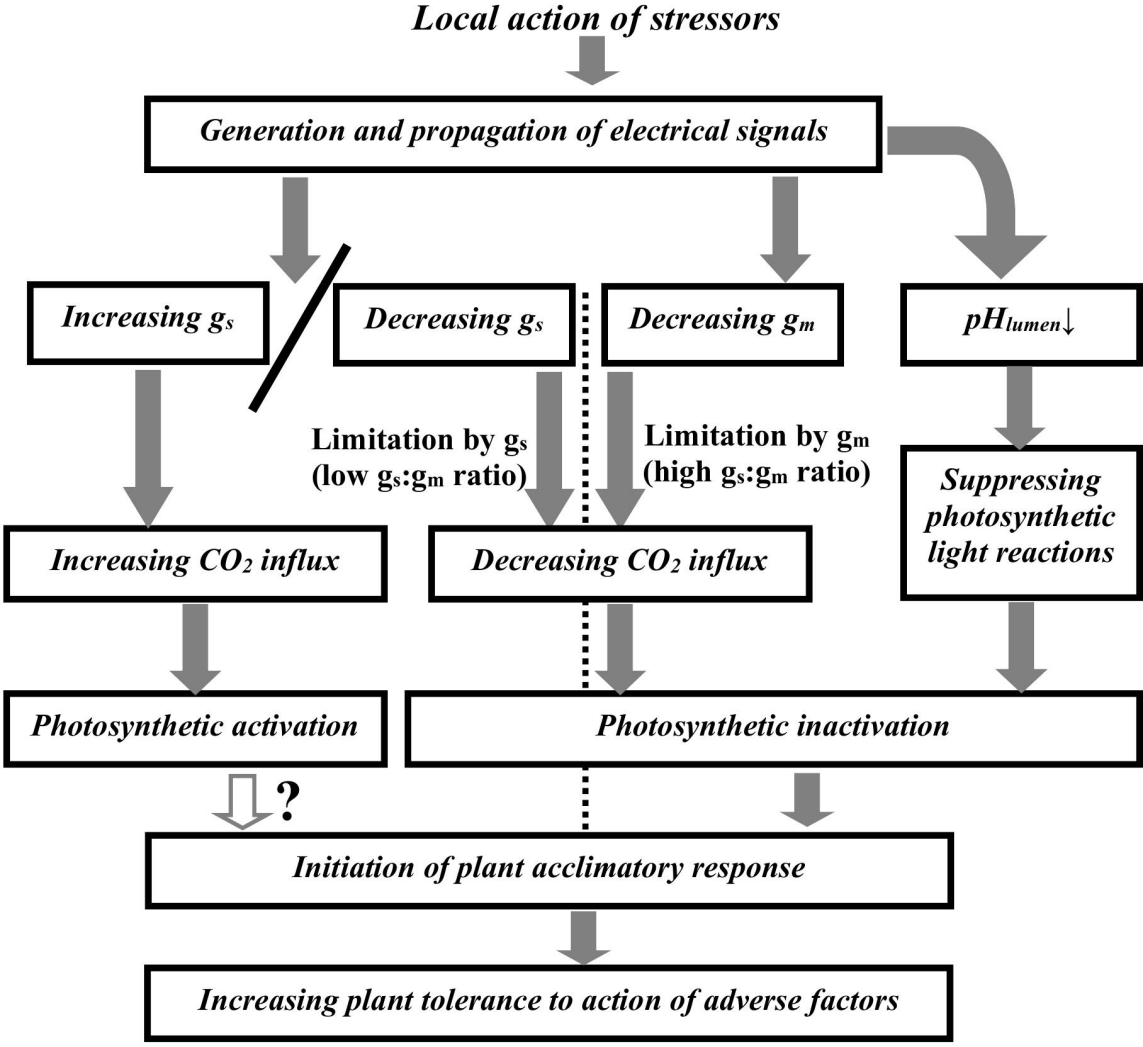
Potential ways of influence of electrical signals on photosynthetic activity. g_s_ is the stomatal CO_2_ conductance, g_m_ is the mesophyll CO_2_ conductance. pH_lumen_ is pH in the chloroplast lumen. In accordance with Equation 1, the low ratio of the initial g_s_ to the initial g_m_ corresponds to limitation of the CO_2_ flux from air to the chloroplast stroma by the stomatal CO_2_ conductance; the high ratio of the initial g_s_ to the initial g_m_ corresponds to limitation of this CO_2_ flux by the mesophyll CO_2_ conductance. Initial conductance is conductance before induction of electrical signals.

The current analysis shows that ESs-induced photosynthetic responses in terrestrial plants can be result of combination of three groups of processes including suppressing photosynthetic light reactions by acidification of the chloroplast lumen, decreasing g_m_, and increasing/decreasing g_s_ (**Figure 1**). The pH-dependent suppression of light reactions is not probable to be strongly related to changes in g_m_ and g_s_. In contrast, changes in the mesophyll and stomatal CO_2_ conductance should strongly influence the CO_2_ flux from air to the chloroplast stroma and, thereby, photosynthesis.

The ratio between initial g_s_ and g_m_ is probable to be the first key criterion to determine parameters of ESs-induced photosynthetic responses. The high g_s_:g_m_ ratio contributes to induction of typical fast photosynthetic inactivation (Sukhov, 2016) which is based on the ESs-induced decreasing g_m_ (Gallé et al., 2013). In this case, dynamics of changes in A_hv_ and other photosynthetic parameters can be weakly related to changes in the stomatal conductance (Koziolek et al., 2004; Kaiser and Grams, 2006; Grams et al., 2009; Gallé et al., 2013).

In contrast, the low g_s_:g_m_ ratio can contribute induction of different types of photosynthetic responses. This type is dependent on the second key criterion which is direction of changes in g_s_. In this case, the ESs-induced decreasing g_s_ should also cause the photosynthetic inactivation; however, dynamics of this inactivation should be strongly related to dynamics of the stomata closure. Particularly, this response was shown in tobacco (Hlavácková et al., 2006) and tomato (Hlavinka et al., 2012) after the local burning and the ESs propagation. The ESs-induced increasing g_s_ should cause the photosynthetic activation with dynamics strongly related to dynamics of stomata opening. This relation is observed in works showing the ESs-induced photosynthetic activation (Grams et al., 2007; Vuralhan-Eckert et al., 2018; Yudina et al., 2022; Grinberg et al., 2023).

It should be additionally noted that proposed mechanism explains the ESs-induced A_hv_ activation, However, increasing the quantum yield of photosystem II can be also observed after the ESs propagation (Grinberg et al., 2023). This effect is probable to be caused by stimulation of ATP consumption and following decrease of electrochemical H^+^ gradient across the thylakoid membrane; this decreasing can stimulate electron flows and increase the quantum yield of photosystem II (Tikhonov, 2013, 2014).

Thus, the current analysis preliminary explains potential mechanisms of the ESs-induced photosynthetic activation which was observed in some works (see above). Further checking this explanation requires experimental and model-based investigations; however, there are other questions related to the ESs-induced photosynthetic activation. Particularly, the ESs-induced photosynthetic inactivation is considered to play an important role in increasing tolerance of plants to action of adverse factors (Sukhov, 2016; Sukhova et al., 2023). Considering this point, analysis of influence of the photosynthetic activation on the plant tolerance is an additional important task of future investigations.
